# Factors Associated With the Actual Behavior and Intention of Rating Physicians on Physician Rating Websites: Cross-Sectional Study

**DOI:** 10.2196/14417

**Published:** 2020-06-04

**Authors:** Xi Han, Bei Li, Tingting Zhang, Jiabin Qu

**Affiliations:** 1 School of Business Administration Guangdong University of Finance & Economics Guangzhou China; 2 School of Health Service Management Southern Medical University Guangzhou China; 3 School of Information Engineering Nanjing Audit University Nanjing China; 4 Library of Yantai University Yantai China

**Keywords:** online physician rating, user-generated content, physician rating website, behavioral intention, actual behavior

## Abstract

**Background:**

Although online physician rating information is popular among Chinese health consumers, the limited number of reviews greatly hampers the effective usage of this information. To date, little has been discussed on the variables that influence online physician rating from the users’ perspective.

**Objective:**

This study aims to investigate the factors associated with the actual behavior and intention of generating online physician rating information in urban China.

**Methods:**

A web-based cross-sectional survey was conducted, and the valid responses of 1371 Chinese health consumers were recorded. Using a pilot interview, we analyzed the effects of demographics, health variables, cognitive variables, and technology-related variables on online physician rating information generation. Binary multivariate logistic regression, multiple linear regression, one-way analysis of variance analyses, and independent samples *t* test were performed to analyze the rating behavior and the intentions of the health consumers. The survey instrument was designed based on the existing literature and the pilot interview.

**Results:**

In this survey, 56.7% (778/1371) of the responders used online physician rating information, and 20.9% (287/1371) of the responders rated the physicians on the physician rating website at least once (posters). The actual physician rating behavior was mainly predicted by health-related factors and was significantly associated with seeking web-based physician information (odds ratio [OR] 5.548, 95% CI 3.072-10.017; *P*<.001), usage of web-based physician service (OR 2.771, 95% CI 1.979-3.879; *P*<.001), health information-seeking ability (OR 1.138, 95% CI 0.993-1.304; *P*=.04), serious disease development (OR 2.699, 95% CI 1.889-3.856; *P*<.001), good medical experience (OR 2.149, 95% CI 1.473-3.135; *P*<.001), altruism (OR 0.612, 95% CI 0.483-0.774; *P*<.001), self-efficacy (OR 1.453, 95% CI 1.182-1.787; *P*<.001), and trust in online physician rating information (OR 1.315, 95% CI 1.089-1.586; *P*=.004). Some factors influencing the intentions of the posters and nonposters rating the physicians were different, and the rating intention was mainly determined by cognitive and health-related factors. For posters, seeking web-based physician information (β=.486; *P*=.007), using web-based medical service (β=.420; *P*=.002), ability to seek health information (β=.193; *P*=.002), rating habits (β=.105; *P*=.02), altruism (β=.414; *P*<.001), self-efficacy (β=.102; *P*=.06), trust (β=.351; *P*<.001), and perceived ease of use (β=.275; *P*<.001) served as significant predictors of the rating intention. For nonposters, ability to seek health information (β=.077; *P*=.003), chronic disease development (β=.092; *P*=.06), bad medical experience (β=.047; *P*=.02), rating habits (β=.085; *P*<.001), altruism (β=.411; *P*<.001), self-efficacy (β=.171; *P*<.001), trust (β=.252; *P*<.001), and perceived usefulness of rating physicians (β=.109; *P*<.001) were significantly associated with the rating intention.

**Conclusions:**

We showed that different factors affected the physician rating behavior and rating intention. Health-related variables influenced the physician rating behavior, while cognitive variables were critical in the rating intentions. We have proposed some practical implications for physician rating websites and physicians to promote online physician rating information generation.

## Introduction

With the development of user-generated content and prevalent use of mobile devices, some industries (ie, food service, travel, and e-commerce) have begun gathering web-based reviews, because of which many websites of these industries have now become reliable and effective [1]. The health care system has also started garnering web-based reviews, even though the development of the review platform was slow in the initial years. Similar to how people review products, health consumers can post their opinions on the health care received and have access to other patients’ opinions on the care they received from physicians. In particular, information on physician rating websites seems to play an increasingly important role in the life of health consumers and has attracted the attention of medical practitioners. A study [2] showed that in 2007, only 3%-7% of the health consumers in the United States used physician rating websites, but the proportion increased to 23% in 2012 [3], 25% in 2013 in Germany [4], 42% in 2014 in the United States [5], and 43.6% in 2016 in Germany [6]. Online physician rating information is an important factor that seems to increasingly influence a patient’s choice of medical practitioners [7]. On the contrary, physicians have always reported a negative attitude toward online physician rating information [8-10] because they fear that limited reviews could produce bias and negative web-based reviews could ruin their reputation [11,12]. Previous studies have shown the content analyses of physician rating websites in different clinical specialties [13-16], and the average number of reviews per physician was found to be low [17-19], even though the number of reviews has increased rapidly in the past few years [20]. Emmert et al [4] reported that only 11.03% of the Germans had posted ratings on a physician rating website in 2013, while this percentage increased to 23% in 2016 [6]. The limited number of reviews is the key factor that has affected the adoption of online physician rating information by both physicians and consumers. Thus, it is important to investigate the factors that predict the generation of online physician rating information from the perspective of health consumers.

Previous studies have mainly focused on the usage of online physician rating information and the related factors. Terlutter et al [21] reported that women, young adults, and people with higher education levels or chronic diseases used physician rating websites more than their counterparts. Galizzi et al [22] found that white British people and people with high incomes were less likely to use physician rating websites. Further, female participants, widows, and those with high health care utilization showed a significantly high likelihood of being aware of physician rating websites [4]. In China, due to the promotion of the “internet + health care” strategy by the government, physician rating websites are becoming increasingly popular among urban citizens. Hao et al [23-25] conducted a content analysis of Chinese physician rating websites and identified factors that were related to physician ratings. Zhang et al [26] analyzed the negative comments on physician rating websites to identify the possible solutions for improving patient satisfaction. Li et al [27] developed a hierarchical topic taxonomy to uncover the latent structure of the physician reviews and illustrated its application in mining data on patients’ interests. Deng et al [28], Han et al [29], and Li and Hubner [30] investigated how web-based ratings and other factors influence the selection of physicians by the Chinese consumers. However, studies on physician rating websites in the Chinese context are still limited and little is known about the factors influencing the generation of online physician rating information.

To fill this research gap, we first conducted a web-based pilot interview and recruited 30 Chinese citizens with different education levels, occupational backgrounds, and hometowns. We introduced several Chinese physician rating websites at the beginning of the interview; thereafter, the participants reported their experience of using the physician rating websites. Only 5 of the 30 participants generated online physician rating information. Finally, participants were asked why they did or did not generate online physician rating information. Following the procedure of qualitative analysis, 3 researchers transcribed and coded the data, and we finally identified the factors related to the generation of online physician rating information. These factors were divided into three dimensions, namely, health and habit-related factors (ie, usage of web-based physician service, ability to seek health information, health conditions, experience of medical service, and rating habits in the e-commerce context), cognitive factors (ie, altruism, self-efficacy, and trust in online physician rating information), and technology-related factors (ie, perceived usefulness and perceived ease of use). Cognitive factors were often reported to be associated with knowledge-sharing behavior [31], and technology-acceptance factors were often associated with system adoption [32]. The use of physician rating websites to rate physicians signifies knowledge-sharing and system-adoption behavior. In this study, we applied a similar procedure used by Terlutter et al [21], Galizzi et al [22], and Emmert et al [4] to empirically explore the significant factors that predict the actual behavior and intention of rating the physicians on the physician rating websites. The results of this study will be useful for understanding the web-based rating behavior of health consumers and for further promoting the development of physician rating websites.

## Methods

### Participant Recruitment

Since physicians in rural areas are seldom rated on physician rating websites [23], our study focused on physicians in the urban regions of China. We used the snowball sampling method to recruit participants through web-based social networking. First, we selected 160 WeChat friends with varying gender, education levels, and occupational backgrounds to complete the web-based questionnaire. Second, we requested these participants to invite friends with varying genders, education levels, and occupational backgrounds to participate in the web-based survey. In total, we received 1556 responses from September 2018 to October 2018 and from August 2019 to October 2019. Among the total number of responders, 185 were excluded from the analysis because of inconsistent answer patterns (eg, flatliners, contradictions) or because the participants tried to complete the questionnaire quickly with incomplete answers in a short span of time. Finally, this study considered the responses of 1371 valid respondents. We paid each participant 2 RMB (US $0.3) to compensate for their time.

### Questionnaire Design

The researchers designed a survey based on the existing literature [21,22] and their pilot interview. All items, except categorical variables, were measured using a 7-point Likert scale, with the options ranging from “strongly disagree” to “strongly agree” (Multimedia Appendix 1). To ensure the validity of the scale in our questionnaire, we adopted measurement items from the existing literature and we modified some items to adapt to the online physician rating scenario based on our first pilot study with 30 Chinese citizens. We calculated the mean values of the multiple items as predictor scores after checking the measurement’s internal reliability.

The questionnaire was created in English. One researcher translated it into Chinese, and then another researcher translated it back into English to ensure the consistency of the content. After developing the Chinese questionnaire, 3 researchers in health informatics were invited to assess the ease of understanding, logical consistencies, item sequence, and contextual relevance of the items in the questionnaire. We made some minor modifications based on their suggestions. Furthermore, a pilot test was conducted with 20 participants, and the items were modified slightly.

### Measurements

#### Rating Behavior, Rating Intention, and Demographic Variables

To ensure that the respondents understood the online physician rating system, a screenshot of a physician rating website was presented in the introductory phase before the respondents answered the questions. The actual behavior of rating a physician was assessed by asking if the respondents had rated physicians on physician rating websites previously (0=no, 1=yes). We defined participants as posters if they had rated a physician on a physician rating website at least once and we defined participants as nonposters if they had never rated a physician on the physician rating website. The intention of rating a physician was assessed using a 3-item scale adapted from the study of Ajzen [33]. This scale was found to be reliable (mean 5.064, SD 1.189; Cronbach α=.949). Additionally, data on demographic variables such as age, gender, education level, marital status, monthly income, daily internet use, and the number of vulnerable family members were also collected (Multimedia Appendix 1).

#### Health and Habit-Related Variables

The usage of web-based physician information was assessed by asking respondents if they had ever sought physician information on the internet (0=no, 1=yes). The usage of web-based physician service was assessed by asking participants if they had ever booked or consulted a physician on the internet (0=no, 1=yes). The ability to seek health information was assessed using 2 items adapted from the model developed by Richard et al [34]. The scale (mean 4.649, SD 1.350; Cronbach α=.890) assessed the participant’s ability to search for web-based health information. The health conditions of the participants or their family members were measured using the following 2 questions: “Did you or your family members develop any chronic disease in the past 2 years?” and “Did you or your family members develop any serious disease in the past 2 years?” (0=no, 1=yes). Medical experience was measured using the following questions: “I had a very good medical experience in the past 2 years” and “I had a very bad medical experience in the past 2 years” (0=no, 1=yes). In the e-commerce era, some consumers have the habit of posting reviews after performing web-based transactions, and the habit was found to be effective in the online physician rating scenario in our pilot interview. The following rating habit in the e-commerce context was assessed using an item adapted from a previous study [35]: “Rating the product after a web-based transaction has become a habit for me” (1=strongly disagree, 7=strongly agree).

#### Cognitive Variables

Altruism is a behavior intended to benefit another, even when this action may involve sacrificing one’s welfare [36]. A 3-item scale on altruism was adapted from previous studies [37,38] and applied in our pilot interview. This scale was found to be reliable (mean 5.438, SD 1.042; Cronbach α=.910). Self-efficacy refers to the belief or the estimate of an individual about his/her own ability to perform a particular task [39]. The self-efficacy scale was adapted from prior studies and applied in our pilot interview [40,41], and the scale was found to be reliable (mean 4.594, SD 1.202; Cronbach α=.770). Trust refers to a situation in which one party willingly relies on the actions of the other party [42]. The scale for trust in online physician rating information was adapted from a previous study [43], and this scale was also found to be reliable (mean 4.711, SD 1.224; Cronbach α=.885).

#### Technology-Related Variables

Perceived usefulness refers to the usefulness of using physician rating websites to rate physicians, and perceived ease of use refers to the ease of using physician rating websites to rate physicians. Based on our pilot interview and previous studies [44,45], we adapted reliable scales for perceived usefulness (mean 5.147, SD 1.162; Cronbach α=.842) and perceived ease of use (mean 4.405, SD 1.127; Cronbach α=.742).

### Data Analysis

Data were downloaded from the web-based questionnaire database to computers in our laboratory in Nanjing University, China. Two independent research assistants examined the data and removed 185 unqualified cases. Data analyses were conducted using the SPSS 23.0 software (IBM Corp). We examined the descriptive statistics for all the variables. Since we focused on participants who were aware of physician rating websites before completing our survey, binary logistic regression analysis was performed to examine the effect of the variables on the likelihood of generating online physician rating information. Multiple linear regression, one-way analysis of variance (ANOVA), and independent samples *t* test (two-tailed) were performed to investigate the different factors influencing the physician rating intentions of the posters and nonposters. We applied data screening procedures to identify problematic patterns within the data set before performing linear regression. Linear relationship, multivariate normality, multicollinearity tested by variance inflation factors (VIFs), autocorrelations tested by Durbin-Watson, and homoscedasticity were tested, and we found that the data could be used for further linear regression. A bootstrapping procedure (with 5000 bootstrap samples) was used in our regression models.

## Results

### Demographic Data of the Participants

The demographic characteristics of the participants are presented in Table 1. The age range of most of the participants was between 25 and 40 years. Out of the 1371 participants, 789 (57.6%) were women and 980 (71.5%) were married. The monthly income of 69.1% (947/1371) of the participants ranged between 3000 RMB (US $435) and 12,000 RMB (US $1740). With respect to the education level, 68.5% (939/1371) of the participants had completed college or higher level of education. Of the 1371 participants, 778 (56.7%) used online physician rating information and 287 (20.9%) rated the physicians on physician rating websites.

**Table 1:** Demographic characteristics of the sample (n=1371).

Demographic characteristics		Value, n (%）
**Age (years)**		
	≤24	160 (11.7)
	25-30	251 (18.3)
	31-35	278 (20.3)
	36-40	242 (17.7)
	41-45	204 (14.9)
	≥46	236 (17.2)
**Gender**		
	Female	789 (57.5)
	Male	582 (42.5)
**Income (RMB ¥)^a^**		
	≤3000	164 (12.0)
	3001-6000	337 (24.6)
	6001-9000	326 (23.8)
	9001-12,000	284 (20.7)
	≥12,001	260 (19.0)
**City level**		
	County/bureau level	337 (24.6)
	Provincial level	402 (29.3)
	Metropolitan	632 (46.1)
**Education**		
	Middle school	432 (31.5)
	Undergraduate	527 (38.4)
	Postgraduate	412 (30.1)
**Marital status**		
	Unmarried	391 (28.5)
	Married	980 (71.5)
**Children and elders**		
	0	240 (17.5)
	1	235 (17.1)
	2	317 (23.1)
	3	209 (15.2)
	4	178 (13.0)
	≥5	192 (14.0)
**Daily internet use**		
	T^b^≤3 h	158 (11.5)
	3<T≤5 h	303 (22.1)
	5<T≤7 h	292 (21.3)
	7<T≤9 h	294 (21.4)
	9<T≤11 h	324 (23.6)
**Online physician rating awareness**		
	Aware	972 (70.9)
	Unaware	399 (29.1)
**Online physician rating usage**		
	Nonusers	593 (43.3)
	Users	778 (56.7)
**Online physician rating generation**		
	Nonposters	1084 (79.1)
	Posters	287 (20.9)

^a^A currency exchange rate of RMB ¥1=US $0.14 is applicable.

^b^T: Time.

### Factors Associated With the Actual Behavior of Rating Physicians on Physician Rating Websites

We focused on participants who were aware of physician rating websites before our survey (n=972), and Table 2 and Table 3 show the results of 4 binary logistic regressions. In the first step, a binary logistic regression between having rated a physician or not (yes=1, no=0) as the criterion and demographic variables was performed (Model 1). Model 1 was significant. Age (β=.146; *P=*.06), monthly income (β=.197; *P=*.003), and education level (β=–.308; *P=*.02) were significantly associated with the likelihood of rating a physician on physician rating websites. However, we also noticed that Nagelkerke *R^2^* (.041) was quite low, and –2 log-likelihood (1130.138) was high. The results indicated that demographic variables only explained a small part of the actual rating behavior.

Then, we entered the health- and habit-related variables into regression Model 2. The model improved with a Nagelkerke *R^2^* change of 0.238. The regression coefficients were significant for the following variables: experience of seeking physician information on the internet (β=1.713; *P<*.001), usage of web-based physician service (β=1.019; *P<*.001), ability to seek health information (β=.129; *P=*.04), development of serious diseases (β=.993; *P<*.001), and good medical experience (β=.765; *P<*.001). We also noticed that gender (β=.410; *P=*.02) and marital status (β=–.441; *P=*.047) were significant factors associated with the actual rating behavior, while age (β=.116; *P=*.16) was not significant after health-related factors were considered.

Following Model 2, cognitive variables were entered into Model 3, which were also significant (*P<*.001). Furthermore, Model 3 showed a minor improvement over Model 2, with increased Nagelkerke *R^2^* change of 0.035. The significant factors in Model 2 mentioned above were still significant. Altruism was negatively (β=–.492; *P<*.001) related to the likelihood of rating physicians. Self-efficacy (β=.374; *P<*.001) and trust in online physician rating information (β=.274; *P=*.004) were significantly and positively related to the likelihood of the rating behavior.

Based on Model 3, technology-related variables were entered into Model 4. However, no improvement was observed in Nagelkerke *R^2^* and the regression coefficients of perceived usefulness (*P=*.42) and perceived ease of use (*P=*.33) were not significant. Significant variables in Model 2 and Model 3 were also significant in Model 4.

Besides the reliability indices mentioned above, collinearity statistics using VIF and tolerance values were tested. The results showed that VIF scores did not exceed 2.479, and tolerance values were not lower than 0.411. According to the criteria proposed by Montgomery and Peck [46] that VIF should be lower than 10 and tolerance value should be more than 0.1, our results indicated that multicollinearity was not a big concern.

**Table 2:** Binary logistic regressions for online physician rating behavior (Model 1-2).

		Model 1^a^				Model 2^b^				
		β	Sig.^c^	OR^d^	95% CI of OR		β	Sig.	OR	95% CI of OR
Constant		–1.267	.002	0.293	—^e^		–3.612	<.001	0.027	—
**Demographic variables**										
	Age	.146	.06	1.138	0.990-1.308		.116	.16	1.123	0.954-1.322
	Gender	.190	.21	1.209	0.896-1.633		.410	.01	1.507	1.076-2.110
	Monthly income	.197	.003	1.218	1.071-1.384		.183	.01	1.201	1.041-1.387
	Marital status	–.257	.21	0.773	0.518-1.155		–.441	.047	0.644	0.411-1.009
	Number of children and elders	.080	.09	1.084	0.985-1.192		.042	.44	1.043	0.938-1.159
	Education level	–.308	.02	0.735	0.564-0.958		–.700	<.001	0.496	0.365-0.674
	Daily internet use	–.046	.40	0.955	0.858-1.063		–.086	.15	0.917	0.815-1.033
**Health- and habit-related variables**										
	Seeking physician information	—	—	—	—		1.713	<.001	5.548	3.072-10.017
	Usage of web-based medical service	—	—	—	—		1.019	<.001	2.771	1.979-3.879
	Health information seeking ability	—	—	—	—		.129	.04	1.138	0.993-1.304
	Chronic disease	—	—	—	—		–.104	.54	0.901	0.646-1.258
	Serious disease	—	—	—	—		.993	<.001	2.699	1.889-3.856
	Good medical experience	—	—	—	—		.765	<.001	2.149	1.473-3.135
	Bad medical experience	—	—	—	—		.267	.13	1.306	0.926-1.843
	Rating habit	—	—	—	—		.028	.59	1.029	0.928-1.140

^a^χ^2^ /Sig.: 27.887 (*df*=7) /<.001; –2 log-likelihood: 1130.138; Nagelkerke *R^2^*: 0.041.

^b^χ^2^ /Sig.: 210.132 (*df*=15) /<.001; –2 log-likelihood: 947.892; Nagelkerke *R^2^*: 0.279.

^c^Sig.: significance probability.

^d^OR: odds ratio.

^e^Not available.

**Table 3:** Binary logistic regressions for online physician rating behavior (Model 3-4).

		Model 3^a^				Model 4^b^			
		β	Sig.^c^	OR^d^	95% CI of OR	β	Sig.	OR	95% CI of OR
Constant		–3.273	<.001	0.038	—^e^	–3.405	<.001	0.033	—
**Demographic variables**									
	Age	.141	.09	1.151	0.976-1.359	.139	.10	1.149	0.972-1.358
	Gender	.395	.02	1.484	1.053-2.092	.408	.02	1.504	1.065-2.123
	Monthly income	.158	.04	1.171	1.009-1.358	.161	.03	1.174	1.011-1.364
	Marital status	–.526	.02	0.591	0.373-0.937	–.527	.03	0.590	0.371-0.940
	Number of children and elders	.019	.73	1.019	0.915-1.136	.019	.74	1.019	0.914-1.135
	Education level	–.763	<.001	0.466	0.338-0.643	–.764	<.001	0.466	0.337-0.645
	Daily internet use	–.068	.27	0.935	0.829-1.054	–.068	.27	0.934	0.828-1.054
**Health and habit-related variables**									
	Seeking physician information	1.810	<.001	6.113	3.355-11.137	1.812	<.001	6.121	3.357-11.159
	Usage of web-based medical service	.951	<.001	2.589	1.838-3.647	.939	<.001	2.559	1.814-3.610
	Health information seeking ability	.147	.04	1.158	1.009-1.330	.150	.04	1.162	1.010-1.336
	Chronic disease	–.111	.52	0.895	0.638-1.256	–.117	.499	0.889	0.633-1.249
	Serious disease	.958	<.001	2.607	1.812-3.751	.979	<.001	2.662	1.845-3.842
	Good medical experience	.800	<.001	2.227	1.515-3.273	.788	<.001	2.200	1.372-2.849
	Bad medical experience	.325	.07	1.384	0.971-1.971	.322	.07	1.380	0.968-1.967
	Rating habit	–.070	.23	0.933	0.831-1.046	–.077	.20	0.926	0.823-1.042
**Cognitive variables**									
	Altruism	–.492	<.001	0.612	0.483-0.774	–.437	.002	0.646	0.489-0.853
	Self-efficacy	.374	<.001	1.453	1.182-1.787	.383	.001	1.466	1.167-1.843
	Trust	.274	.004	1.315	1.089-1.586	.263	.007	1.301	1.073-1.577
**Technology-related variables**									
	Perceived usefulness	—	—	—	—	–.102	.42	0.903	0.703-1.160
	Perceived ease of use	—	—	—	—	.087	.33	1.091	0.917-1.298

^a^χ^2^ /Sig.: 240.174 (*df*=18) / <.001; –2 log-likelihood: 917.851; Nagelkerke *R^2^*: 0.314.

^b^χ^2^ /Sig.: 241.789 (*df*=20) / <.001; –2 log-likelihood: 916.236; Nagelkerke *R^2^*: 0.316.

^c^Sig.: significance probability.

^d^OR: odds ratio.

^e^Not available.

### Predictive Factors for the Intention of Rating a Physician on Physician Rating Websites

To investigate how the variables influence the rating intention of the participants differently, we divided the samples into 2 groups, namely, posters group and nonposters group. Using hierarchical multiple regression analyses, we tested the effects of different dimensions of the factors on the rating intention. By controlling the demographic variables, we found that health-, cognitive-, and technology-related variables explained 21.3%, 38.1%, and 5.5% of the increased variance in the rating intention of the posters and 12.8%, 48.1%, and 0.4% of the increased variance for nonposters, respectively. The VIF and tolerance values showed that multicollinearity was not a concern in any model.

Table 4 displays the final models. For posters who rated the physicians on the physician rating websites, health and habit-related variables, that is, seeking physician information on the internet (β=.486; *P=*.007), using web-based medical services (β=.420; *P=*.002), ability to seek health information (β=.193; *P=*.002), and habits of ratings (β=.105; *P=*.02) were found to be significantly and positively related to the rating intention. The cognitive variables, that is, altruism (β=.414; *P<*.001), self-efficacy (β=.102; *P=*.06), and trust in online physician rating information (β=.351; *P<*.001) were also significant predictors of the rating intention. Perceived usefulness was not significantly associated with the rating intention (β=–.031; *P=*.63), while perceived ease of use (β=.271; *P<*.001) was a significant predictor.

For nonposters who did not rate the physicians on the physician rating websites, usage of web-based medical service (β=.077; *P=*.003), development of chronic disease (β=.092; *P=*.06), bad medical experience (β=.047; *P=*.02), and habits of ratings (β=.085; *P<*.001) were found to be significantly associated with the rating intention. Similar to that observed in the posters group, altruism (β=.411; *P<*.001), self-efficacy (β=.171; *P<*.001), and trust (β=.252; *P<*.001) were also found to be the predictors of the rating intentions of nonposters. Since nonposters did not post web-based physician reviews, perceived ease of use (β=.017; *P=*.505) was not significantly associated with the rating intention, but perceived usefulness (β=.109; *P=*.001) was a significant predictor of the rating intention.

**Table 4:** Linear regressions of the rating intentions of posters and nonposters.

		Posters^a^ (n=287)					Nonposters^b^ (n=1084)			
		β	Sig.^c^	95% CI		β		Sig.	95% CI	
				Lower	Upper				Lower	Upper
Constant		–.636	.045	–1.258	–0.014	.129		.49	–0.238	0.496
**Demographic variables**										
	Age	–.091	.03	–0.172	–0.010	.015		.51	–0.030	0.061
	Gender	.148	.08	–0.018	0.313	–.004		.93	–0.099	0.090
	Monthly income	.018	.64	–0.056	0.091	–.005		.80	–0.045	0.034
	Marital status	.004	.90	–0.052	0.059	.016		.37	–0.019	0.050
	Number of children and elders	.180	.13	–0.054	0.414	–.063		.31	–0.184	0.058
	Education level	–.016	.56	–0.070	0.038	–.004		.81	–0.034	0.026
	Daily internet use	.010	.89	–0.142	0.162	–.004		.93	–0.091	0.083
**Health- and habit-related variables**										
	Seeking physician information	.486	.007	0.134	0.838	–.075		.14	–0.173	0.024
	Usage of web-based medical service	.420	.002	0.157	0.684	.030		.53	–0.065	0.126
	Health information seeking ability	.193	.002	0.071	0.316	.077		.003	0.025	0.128
	Chronic disease	–.013	.87	–0.168	0.142	.092		.06	–0.004	0.188
	Serious disease	.036	.65	–0.122	0.194	.015		.86	–0.156	0.187
	Good medical experience	–.005	.96	–0.208	0.198	.000		.99	–0.094	0.093
	Bad medical experience	.034	.68	–0.124	0.191	.047		.02	0.021	0.232
	Rating habit	.105	.02	0.018	0.193	.085		<.001	0.055	0.114
**Cognitive variables**										
	Altruism	.414	<.001	0.294	0.535	.411		<.001	0.349	0.474
	Self-efficacy	.102	.061	–0.005	0.208	.171		<.001	0.116	0.225
	Trust	.351	<.001	0.260	0.441	.252		<.001	0.202	0.301
**Technology-related variables**										
	Perceived usefulness	–.031	.635	–0.159	0.097	.109		.001	0.047	0.171
	Perceived ease of use	.275	<.001	0.199	0.350	.017		.505	–0.032	0.066

^a^*F* /Sig.: 123.812 (*df*=20)/ <.001; Nagelkerke *R^2^*: 0.617.

^b^*F* /Sig.: 114.296 (*df*=20)/ <.001; Nagelkerke *R^2^*: 0.623.

^c^Sig.: significance probability.

Furthermore, we used one-way ANOVA and independent samples *t* test to compare the differences between posters (n=287) and nonposters (n=1084) on the rating intention and related factors. Following the suggestion by Fritz et al [47], Cohen *d* was used to estimate the effect size. It can be seen from Table 5 that posters had a higher level of rating intention than nonposters (*t_1369_*=5.569; *P<*.001). Regarding self-efficacy, the 2 groups differed as expected (*t_1369_*=5.771; *P<*.001), with posters ascribing higher self-efficacy than the nonposters. Table 5 also indicated that posters trusted the information on physician rating websites to a greater extent than nonposters (*t_1369_*=5.549; *P<*.001). The 2 groups did not differ significantly in altruism at *P<*.05 level (*t_1369_*=1.697; *P=*.09). Additionally, posters perceived higher levels of usefulness (*t_1369_*=3.020; *P=*.003) and ease of use (*t_1369_*=3.928; *P<*.001) than nonposters. With regard to the effect size, a Cohen *d* value of 0.2 indicated a small effect and a value of 0.5 indicated a medium effect. Thus, the effect sizes for rating intention, self-efficacy, and trust were found to be medium, while the effect sizes for perceived usefulness and perceived ease of use were found to be small.

**Table 5:** Differences between posters and nonposters in the rating intention and related factors.

Variables	Poster, mean (SD)	Nonposter, mean (SD)	*t (df)*	*P* value	Cohen *d*
Rating intention	5.495 (1.120)	5.064 (1.189)	5.569 (1369)	<.001	0.373
Altruism	5.556 (1.074)	5.438 (1.042)	1.697 (1369)	.09	0.112
Self-efficacy	5.052 (1.229)	4.594 (1.202)	5.771 (1369)	<.001	0.377
Trust	5.150 (1.058)	4.711 (1.224)	5.549 (1369)	<.001	0.358
Perceived usefulness	5.380 (1.150)	5.147 (1.162)	3.020 (1369)	.003	0.202
Perceived ease of use	4.700 (1.085)	4.405 (1.127)	3.928 (1369)	<.001	0.267

## Discussion

### Principal Findings

Previous studies on online physician rating information mainly focused on the usage of online physician ratings and related factors [2-4,21], and only 2 studies [4,6] have shown the proportion (11% and 23%) of people who rated the physicians on physician rating websites. Our study focused on the urban Chinese population and found that 20.9% (287/1371) of the respondents rated the physicians on physician rating websites at least once. An important aspect of our study was that we investigated the factors that predicted the actual behavior of rating the physicians on the physician rating websites. Since only 56.7% (778/1371) of the participants had used online physician rating information, we examined the effects of different factors on the rating intentions of the posters and nonposters. Our results also show that the factors affecting the actual rating behavior and rating intention were different, even though the rating behavior was positively related to the rating intention in our partial correlation analysis (*r*=.148; *P<*.001).

Our study shows that only sociodemographic variables cannot produce a satisfying model to predict the actual behavior of rating physicians on the physician rating websites. Even though monthly income and education level were significantly correlated with the rating behavior (Model 1 in Table 2), the Nagelkerke *R^2^* (0.041) of the logistic regression model was low. We also found that gender and marital status were significantly associated with the rating behavior when health and cognitive variables were included. The change in Nagelkerke *R^2^* indicated that it was necessary to integrate additional health and cognitive variables to predict the rating behavior to a more satisfying extent. Health-related factors played an important role in predicting the likelihood of the rating behavior. In our study, participants with the experience of seeking physician information on the internet, who used web-based physician service, and who had higher ability to seek health information were more prone to rate physicians on the physician rating websites. Since there have been incidents of poor physician-patient relationships and severe cases of vicious attacks on medical professionals particularly in China [48], many health consumers choose to check physician information on the internet and seek web-based health information to avoid unpleasant medical experiences. Seeking web-based health information increased their awareness of the online physician rating information and motivated them to rate physicians. Development of serious diseases and good medical experience were also predictors of the rating behavior. This result corroborated that of previous studies that showed a large number of positive reviews on physician rating websites [49-52]. Further, altruism was negatively related to the rating behavior, indicating that egoistic motivation played a role, and nonposters showed exaggerated level of altruism in their behavior of generating online physician rating information. Self-efficacy reflects an optimistic self-belief that one can perform a task, and it was found to be positively related to the rating behavior. In a web-based context, trust is always a big concern, and it was found to be positively related to the usage of online physician rating information [21]. In our study, trust in online physician rating information was also positively related to the rating behavior. However, as most participants had not used physician rating websites, perceived usefulness and perceived ease of use were not significantly associated with the rating behavior.

Regarding the rating intention, cognitive factors explained the largest variance in the rating intention, and factors influencing the rating intention of posters and nonposters were different. The common factors were health information-seeking ability, rating habit, altruism, self-efficacy, and trust in online physician rating information. However, most health- and technology-related variables that predicted the rating intentions of the posters and nonposters were different. For health-related variables, the rating intention of the posters was mainly predicted by the usage of web-based health information or service, while the rating intention of the nonposters was associated with the health status and medical experience. Although the results in Table 2 indicated that serious disease development and good medical experience predicted the actual rating behavior, our linear regression model demonstrated that chronic disease and bad medical experience were associated with the rating intentions of the nonposters after they became aware of physician rating websites. Additionally, perceived usefulness was associated with the rating intention of the nonposters and perceived ease of use was associated with the rating intention of the posters. Further, we noticed that the posters judged the rating intention, altruism, trust in online physician rating information, perceived usefulness, and perceived ease of use higher than the nonposters.

### Practical Implications

Based on the results in our study, we have some recommendations for physician rating websites and physicians who are the stakeholders of online physician rating information generation. Commercial physician rating websites are the main sources for health consumers to access online physician rating information; thus, a large amount of online physician rating information is necessary and critical for the development of physician rating websites. Large amount of online physician rating information can be generated as follows. First, physician rating websites need to cooperate with widely used search engines and social media to increase the awareness of these websites among health consumers, since our results indicated that many consumers were unaware of these websites, and usage of web-based physician information could improve online physician rating information generation behavior and rating intention. Although Chinese physician rating websites have provided services for many years and are top-ranked in the search engine results page, most health consumers are still unfamiliar with these physician rating websites and are uncertain about their reliability. Second, physician rating websites need to cooperate with hospitals officially. Health consumers have high levels of trust in public hospitals. Thus, cooperation with hospitals would enhance consumers’ trust and improve the usability of commercial physician rating websites. Our findings suggested that trust was positively related to the physician rating behavior and intention. In fact, reviews on some physician rating websites in China increased greatly after physician rating websites provided booking services for hospitals. These physician rating websites are becoming increasingly popular among health consumers in cities. Third, physician rating websites must provide additional incentives for health consumers to generate online physician rating information. Knowledge sharing is an altruistic behavior. However, our results indicated altruism to be negatively related to the actual rating behavior. Egoism may play an important role in the actual rating behavior. Thus, a better incentive mechanism is needed for attracting health consumers to rate physicians on the physician rating websites. Fourth, physician rating websites need to cooperate with physicians and provide web-based medical services, besides online physician rating information. In the past 2 years, many physicians in China have begun to use physician rating websites to provide web-based medical services, which have greatly increased the number of reviews and the usability of these websites.

The results of our study could also be interesting for physicians. Online physician rating information is important for physicians to boost their reputation and to enjoy success in their careers. Thus, physicians need to actively encourage patients to generate online physician ratings by performing the following measures. First, physicians should be concerned about patients’ medical experiences. We found that good medical experience predicted the actual behavior of rating the physician on the physician rating websites. This finding is consistent with that reported in previous studies that showed positive reviews for physicians [20,48]. Physicians need not worry about negative reviews ruining their reputation, even though a bad medical experience was positively related to the rating intention of nonposters. Physicians are encouraged to show empathy to their patients, who may consequently provide positive reviews about them. Second, physicians should recommend physician rating websites to their patients and encourage them to provide online physician ratings after receiving the medical service. Physician recommendations would increase patients’ trust in online physician rating information and directly lead to the generation of more reviews. Even though it is embarrassing to be rated by patients, physicians should accept that online physician rating information could lead to their medical service improvement.

### Limitations and Future Direction

This study has some limitations. First, we used a snowball sampling method and focused on well-educated people who were younger than 46 years in China. There is a possibility of selection bias among respondents, even though they are the potential online physician rating information users. Thus, a large randomized sample would certainly be desirable in future studies. Second, we only tested the altruistic motivation, which was found to be negatively related to the rating behavior. Future studies should analyze how egoistic motivation directly affects the rating behavior. Third, we did not differentiate people with bad medical experience from people with good medical experience. Medical experience could be an interesting variable to focus on, considering the special patient-physician relationship in China. Researchers should explore if the kind of medical experience has nuanced the effect on the intention to post online physician rating information with regard to the unsatisfying physician-patient relationships in China. Finally, factors influencing the actual behavior and intention of rating physicians were quite different in our study. Since many participants were unaware of physician rating websites before our survey, it would be better to examine how these factors affect their actual rating behavior. Even though the intention is predictive of future behavior, the self-reported intention might be exaggerated. A long-term follow-up study is needed in the future to investigate how different factors affect the actual rating behavior after health consumers become aware of the online physician rating information.

### Conclusion

Since the limited number of web-based reviews greatly hampers the effective usage of physician rating information, it is important to discuss the variables that influence the generation of physician rating information from the health consumer’s perspective. Our cross-sectional study shows that factors affecting the physician rating behavior and rating intention are different. We found that health-related variables influenced the physician rating behavior while cognitive variables were critical in the rating intentions. Based on our findings, we have provided some practical suggestions for physician rating websites and physicians to promote the generation of online physician rating information.

## Appendix

Multimedia Appendix 1 Survey questionnaire.

## Abbreviations

ANOVA: analysis of variance
VIF: variance inflation factor
