# Supplementary material for: Factors Associated With the Actual Behavior and Intention of Rating Physicians on Physician Rating Websites: Cross-Sectional Study
Source: J Med Internet Res. 2020 Jun 4;22(6):e14417. doi: 10.2196/14417 (PMC7303836; doi:10.2196/14417)
Supplement: Multimedia Appendix 1 [file jmir_v22i6e14417_app1.docx]

**Multimedia Appendix 1: Survey Questionnaire**


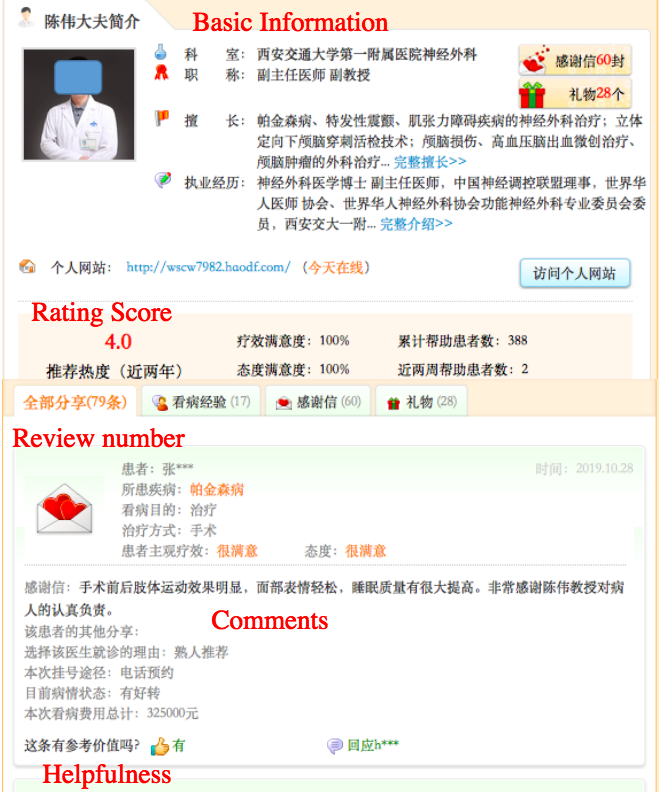


The picture is a screen-shot of the most famous PRW in China.

1.Age

A ≤ 24 B 25-30 C 31-35 D 36-40 E 41-45 F ≥46

2.Gender

A Male B Female C Others

3. Highest education level

A Junior middle school B Senior middle school C College/Bachelor

D Master/Doctor

4. Marital status

A Single B Married C Others

5.Your monthly income (RMB¥)

A ≤3000 B 3001-6000 C 6001-9000 D 9001-12000 E 12001-15000 F15001-18000 G18001-21000 H ≥21001

6. Hours spent on the Internet daily

A T ≤3h B 3<T≤5h C 5<T≤7h D7<T≤9h E 9<T≤11h

F 11<T≤13h G T>13h

7.How many children (less than 8-year-old) and elders (more than 65-year-old) live in your home?

A 0 B 1 C 2 D 3 E 4 F≥5

8.What is your living city level?

A County level B Bureau level C Provincial Level D Metropolitan

9.Did you or your family members develop any chronic disease in the past 2 years?

A No B Yes

10. Did you or your family members develop any serious disease in the past 2 years?

A No B Yes

11.Have you ever sought physicians information online?

A No B Yes

12.Have you ever booked or consulted a physician online?

A No B Yes

13.I have had a very good medical experience in the past 2 years.

A No B Yes

14.I have had a very bad medial experience in the past 2 years.

A No B Yes

15.Were you aware of PRWs or online physician rating information before our survey?

A No B Yes

16.Have you ever sought /used online physician rating information?

A No B Yes

1. Have you ever rated or commented a physician on PRWs?

A No B Yes

1. Your agreement with the following items: (1-7: strongly disagree to strongly agree)

| Variable | Items | 1 | 2 | 3 | 4 | 5 | 6 | 7 |
| --- | --- | --- | --- | --- | --- | --- | --- | --- |
| **Health information seeking ability**  [34] | I am skilled at seeking online health information. |  |  |  |  |  |  |  |
|  | I am considering myself very knowledgeable about online health information search techniques. |  |  |  |  |  |  |  |
| **Habit**[35] | Rating the product after an web-based transaction has become a habit for me. |  |  |  |  |  |  |  |
| **Altruism**[37,38] | I want to benefit other patients by rating physician online. |  |  |  |  |  |  |  |
|  | I am willing to help others to make medical decisions by posting my own medical experience on PRWs. |  |  |  |  |  |  |  |
|  | I enjoy helping others to know more about the physician by  posting comments on PRWs. |  |  |  |  |  |  |  |
| **Self-efficacy**[40,41] | I have confidence in my ability to provide online physician reviews that users consider valuable. |  |  |  |  |  |  |  |
|  | I am confident that if I want, I can rate or comment a physician on a PRW. |  |  |  |  |  |  |  |
|  | I am sure that I have time and opportunities to rate or comment a physician on a PRW. |  |  |  |  |  |  |  |
| **Trust**[43] | I believe other patients’ experience on PRWs would be reliable. |  |  |  |  |  |  |  |
|  | I believe online rating scores or comments by other patients would be dependable. |  |  |  |  |  |  |  |
|  | I think rating information on PRWs by patients would be trustworthy. |  |  |  |  |  |  |  |
| **PU**[44,45] | I think rating physician on PRWs would promote physician to provide better service for me. |  |  |  |  |  |  |  |
|  | I believe rating physician on PRWs would express my compliments or dissatisfaction. |  |  |  |  |  |  |  |
|  | I think rating physician on PRWs would improve the medical service I enjoy. |  |  |  |  |  |  |  |
| **PEOU**[44,45] | It is easy for me to rate physician on PRWs. |  |  |  |  |  |  |  |
|  | Learning to comment a physician on PRWs is easy. |  |  |  |  |  |  |  |
|  | It is easy to become skillful in rating physician on PRWs. |  |  |  |  |  |  |  |
| **Rating intention**[33] | I think I will rate physician on PRWs in the future. |  |  |  |  |  |  |  |
|  | I intend to post reviews on PRWs someday. |  |  |  |  |  |  |  |
|  | I plan to comment physician on a PRW. |  |  |  |  |  |  |  |
